# Arc of Buhler: A lifesaving anatomic variation. A case report

**DOI:** 10.1590/1677-5449.200045

**Published:** 2020-11-23

**Authors:** Schizas Nikolaos, Patris Vasilios, Lama Niki, Eleftherios Orestis Argyriou, Kratimenos Theodoros, Argiriou Mihalis

**Affiliations:** 1 Evangelismos General Hospital of Athens, Department of Cardio Thoracic and Vascular Surgery, Athens, Greece.; 2 Kapodistrian University of Athens, Research Unit of Radiology and Medical Imaging, Greece.; 3 Whipps Cross University Hospital, Barts Health NHS Trust, Departement of Colorectal Surgery, London, UK.; 4 Evangelismos General Hospital of Athens, Interventional Radiology Unit, Department of Radiology, Athens, Greece.

**Keywords:** complicated type B aortic dissection, visceral malperfusion, celiac artery, mesenteric artery, TEVAR, dissecção aórtica do tipo B complicada, má perfusão visceral, artéria celíaca, artéria mesentérica, TEVAR

## Abstract

The presence of malperfusion syndrome in cases of complicated acute type B aortic dissection is a negative predictive factor and urgent intervention is indicated. Anatomic variations, such as the Arc of Buhler, contribute anastomotic channels and can preserve the visceral blood supply. In this case report, we describe the overall management of a 54-year-old man who presented with a type B aortic dissection. Initially, conservative management was chosen, as indicated for an uncomplicated type B dissection, but the dissection deteriorated. Despite the fact that severe occlusion of the celiac artery was detected on Computed Tomography (CT) angiography, the Arc of Buhler anatomical variation was present, contributing adequate visceral blood supply. After considering this finding, the patient was treated effectively with thoracic endovascular aortic repair (TEVAR).

## Resumo

A presença da síndrome de má perfusão em casos de dissecção aórtica aguda do tipo B complicada é um fator preditor negativo, e uma intervenção urgente é indicada. As variações anatômicas, tais como o arco de Buhler, contribuem como canais anastomóticos e podem preservar o suprimento sanguíneo visceral. Neste relato de caso, descrevemos o manejo geral de um paciente do sexo masculino, de 54 anos, que apresentou uma dissecção aórtica do tipo B. Inicialmente, o manejo conservador foi escolhido, conforme indicado para dissecção do tipo B não complicada, mas a dissecção sofreu deterioração. Apesar de uma oclusão grave da artéria celíaca ter sido detectada na angiotomografia computadorizada, a variação anatômica do arco de Buhler estava presente, contribuindo para o suprimento sanguíneo visceral adequado. Após levar em consideração esse achado, o paciente foi tratado de forma efetiva com reparação endovascular da aorta torácica.

## INTRODUCTION

Malperfusion syndrome is defined as reduced blood supply to a vital organ caused by branch arterial obstruction secondary to the dissection.^1^ Malperfusion provokes end-organ ischemia and its progression results in organ failure. Malperfusion syndrome in patients with aortic dissection, type A or type B, is a common cause of major morbidity or death. In the IRAD study (International Registry of Acute Aortic Dissection), mesenteric ischemia was found to be the second most frequent cause of death in patients with type A aortic dissection.^2^ Furthermore, mortality rates in patients with mesenteric malperfusion due to dissection range from 70% to 100%.^3^^,^^4^ This body of evidence suggests that diagnosis of visceral malperfusion is very important and can save lives. On the other hand, the type and the time of the procedure remain controversial in the field of complicated type B aortic dissection.

In this case report, we describe overall management of a 54-year-old man who presented with a type B aortic dissection. Initially, conservative management was chosen, as indicated for an uncomplicated type B dissection, but the dissection deteriorated. Despite the fact that a severe occlusion of the celiac artery was detected on Computed Tomography (CT) angiography, the Arc of Buhler anatomical variation was present and provided adequate visceral blood supply. After taking this finding into consideration, the patient was treated effectively with thoracic endovascular aortic repair (TEVAR).

## CASE REPORT

**Figure 1:**
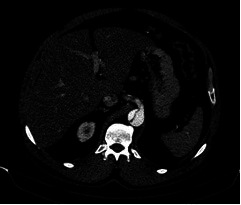
CT angiography image of dissection of the Celiac trunk, as a consequence of a type B aortic dissection.

**Figure 2:**
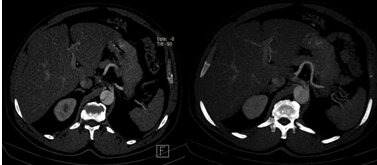
Images of the type B aortic dissection and Celiac trunk dissection. Both branches of the splenic artery are patent.

A 54-year-old man presented at emergency with a complaint of sudden, severe, and generalized abdominal pain. The pain was persistent without any radiation mentioned from the patient. The heart echo and the laboratory tests were normal. Computed tomography angiography (CTA) showed a type B aortic dissection, originated distally of the left subclavian artery and extended to the left iliac artery. The celiac trunk was also dissected, while the superior mesenteric and both renal arteries were supplied by the true lumen (Figure 1 and 2).

A conservative management strategy was chosen, with administration of anti-hypertensive treatment and in-hospital observation. The patient’s condition remained stable and on the fifth day after diagnosis he was discharged from hospital and endovascular restoration of the type B dissection was scheduled for 2 weeks later, in a sub-acute phase.

**Figure 3:**
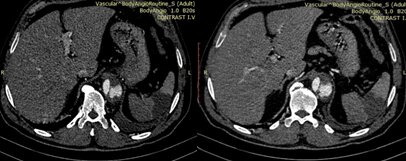
CT angiography 10 days after presentation: Type B aortic dissection and Celiac trunk dissection. One of the branches of the splenic artery is no longer patent, and there is an obvious splenic infarct.

After four days, the patient presented again, with generalized abdominal pain and two episodes of vomiting containing food material. An emergency CT angiography was performed, showing that the type B aortic dissection was similar, but the Celiac trunk dissection had aggravated, although blood supply was preserved through the hepatic and left gastric arteries. One of the branches of the splenic artery was no longer patent, and a splenic infarct was detected (Figure 3). There were no differences in laboratory test results compared to the previous admission.

**Figure 4:**
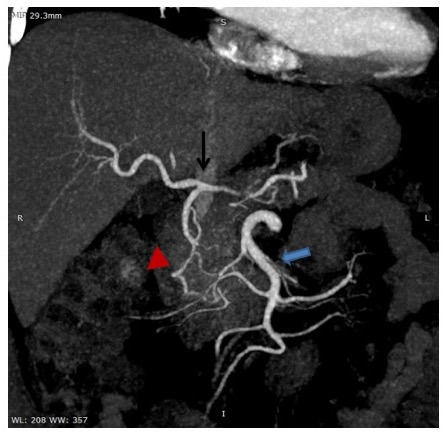
CT angiography image on which the main branches are marked. Superior Mesenteric Artery (thick blue arrow), Common Hepatic Artery (thin black arrow), Arc of Buhler (red arrow head). Although the celiac trunk is almost occluded, blood flow to organs is preserved by the Arc of Buhler.

An urgent intervention decision was taken, in order to avoid organ ischemia. Careful observation of the CT angiography identified the Arc of Buhler anatomical variation (Figure 4). This random finding suggested that the blood supply could be maintained, since the flow from superior mesenteric artery was intact. Taking into consideration this fact, we decided to reject the option of a bypass of the celiac trunk but the intervention through Thoracic Endovascular Aortic Repair (TEVAR) couldn’t be postponed, as the dissection was progressing. A 43 x 37 x 200mm stent graft (LifeTech endograft) was placed distally to the left subclavian artery, covering the entry tear, while its distal edge was proximal to celiac trunk. Despite the fact that the celiac trunk was occluded to an important degree, blood supply to the organs was maintained with absence of clinical deterioration due to malperfusion.

Postoperative recovery was uneventful and the patient was discharged from hospital after 6 days. The results of CT angiography after one month were satisfactory enough, but unfortunately he died in a car accident 2 months later.

## DISCUSSION

Complicated aortic type B dissection is an extremely life-threatening situation, as the perioperative mortality ranges from 36% to 60%.^5^ Early diagnosis of malperfusion syndrome is fundamental and is based on meticulous evaluation of clinical signs and symptoms and laboratory and imaging tests. Moreover, ischemia of the organs due to decreased blood flow is associated with poor surgical outcomes and may present at various stages (immediately after the dissection, during thrombosis of the false lumen causing branch occlusion, at implementation of cardiopulmonary bypass, after completion of aortic restoration).^6^ According to the literature, urgent intervention is indicated when there is a high clinical or imaging suspicion of possible catastrophic complications, such as organ malperfusion, regardless of the surgical approach preferred (open surgery or minimal invasive techniques).^5^ In our case, the progression of the dissection and the presence of almost total occlusion of the celiac artery combined with clinical symptoms (vomiting) suggested that any delay of the procedure could be fatal.

Another key point in the management of this case is planning of the operation and especially the preservation of visceral blood supply. Although, there are collateral pathways between the celiac and superior mesenteric arteries, evaluation of the anastomotic channels is indispensable before any endovascular manipulation of the paravisceral aorta.^7^ The celiac artery and the superior mesenteric are connected through the pancreaticoduodenal arcade which is formed by the superior and inferior pancreaticoduodenal arteries.^8^ At this point, we have to mention that the increased blood flow through the pancreaticoduodenal artery after occlusion, obstruction or coverage of the celiac artery is correlated with enhanced risk of aneurysm formation in this vessel.^9^ Anatomic variations found inconstantly, such as the Arc of Buhler and the Arc of Barkow, are also collateral pathways between the celiac and mesenteric arteries.^8^ Collateral pathways should be evaluated in all cases of celiac artery obstruction. Luckily, in this case, the presence of the Arc of Buhler augmenting the anastomotic channels between the two arteries was a positive predictor of adequate blood supply to organs. On one hand, any effort to bypass the celiac artery or restore the occlusion would have notably increased both the risk of operating and the extent of the procedure. On the other hand, if the anastomotic channels were limited, an inadequate blood supply could lead to malperfusion. Therefore, the presence of the Arc of Buhler was a guarantee that a celiac artery bypass was unnecessary and we could proceed to TEVAR with safety.

At this point, we have to mention that TEVAR is the preferred method for treating complicated type B aortic dissections compared to open surgery. However, the mortality rate ranges between 12.2% and 17% when the procedure is performed in the acute phase.^10^^,^^11^ Aortic rupture is not a rare complication intraoperatively or in the early postoperative period, reaching 7%,^10^ while rates of retrograde type A aortic dissection range from 2 to 10%.^12^ New evidence shows that amelioration of some technical aspects, such as through use of composite device design in patients suffering from acute type B dissection, can lead to reduced mortality rate, although complications and reoperations were similar when compared to stent grafts alone.^11^

## CONCLUSIONS

Complicated type B aortic dissection is a fatal situation, especially when malperfusion syndrome is present. Early diagnosis and intervention are fundamental in these patients. Additionally, the vasculature must be evaluated meticulously when a main branch of the aorta is obstructed or covered. Anatomic variations such as the Arc of Buhler contribute as anastomotic channels and should not be overlooked. TEVAR is an alternative approach to treating acute complicated type B dissection with satisfactory results and a lot of progress is expected over the years to come.
